# Supplementary material for: Outcomes in asymptomatic, severe aortic stenosis
Source: PLoS One. 2021 Apr 7;16(4):e0249610. doi: 10.1371/journal.pone.0249610 (PMC8026050; doi:10.1371/journal.pone.0249610)
Supplement: Attachment — Submitted filename: revision asintomatic severe AS.pdf [file pone.0249610.s001.pdf]

Patient characteristics and outcomes in asymptomatic, severe aortic stenosis

In the present paper Kvaslerud and Colleagues attempt to compare natural history of asymptomatic patients with severe aortic stenosis submitted to a conservative treatment with symptomatic severe aortic stenosis treated with aortic valve replacement. They reviewed medical records of every patient given the ICD-10-code for aortic stenosis (I35.0) at Oslo University Hospital between Dec 2002 and Dec 2016. Patients who were evaluated by the heart team due to severe aortic stenosis were categorized by treatment strategy; they recorded baseline data, adverse events and survival in the group of asymptomatic patients and in a comparison group of 100 age and gender matched patients scheduled for aortic valve replacement (AVR). Authors found that in 2341 patients who were evaluated for aortic valve replacement due to severe aortic stenosis, 114 patients received conservative treatment due to a lack of symptoms. Asymptomatic patients had higher mortality than patients who had AVR. Survival at 1, 2 and 3 years for the asymptomatic patients was 88%, 75% and 63%, compared with 92%, 83% and 78% in the matched patients scheduled for AVR. Age, previous history of coronary artery disease and N-terminal pro B-type natriuretic peptide (NT-proBNP) were predictors of mortality while coronary artery disease and NT-proBNP were predictors of 3-year morbidity in asymptomatic patients.

The optimal timing of AVR in patients with severe aortic stenosis remains a commonly encountered clinical challenge in cardiovascular medicine. Practice guidelines give AVR a class I recommendation in patients with severe aortic stenosis and symptoms of heart failure, angina, or syncope. However, watchful waiting for symptoms is associated with its own risks: sudden death is estimated to affect 1%/y of asymptomatic patients with isolated severe aortic stenosis. Some patients moreover may not be recognized symptoms as related to aortic stenosis; even the straightforward presence of symptoms, especially in the elderly patient, may be difficult to ascertain.

With these considerations, guidelines have added a few additional circumstances in which AVR is reasonable for the asymptomatic patient with severe aortic stenosis; these include left ventricular dysfunction, (defined as ejection fraction <50%), cardiac surgery for other indications, reduced exercise capacity or exertional hypotension demonstrated by exercise testing, or “very severe” aortic stenosis. With the limit of ruling out these recommendations, the paper deals with a very interesting topic, although Authors are to be commended for their attempt to limit the influence of potential confounders.

Major points

- Authors should make a wider description of methods; in a retrospective study evaluating the comparison between two different strategies, watchful waiting and surgical approach, characterization of patients must be more detailed, in particular in table 1 (baselines characteristics) should be inserted comorbidities (such as chronic obstructed pulmonary disease, cancer, chronic renal disease...) and frailty. Recent studies have demonstrated frailty and other geriatric syndromes can identify the most vulnerable patients, thus allowing to better estimate the individual prognosis in terms of disability, readmission and mortality. Such information should also be considered in the decision-making process, ensuring the benefit derived from each treatment in each patient, optimizing the resources and avoiding futility. In the present study a not negligible percentage, 15.7% of patient population, developed symptoms during follow-up, but were considered too comorbid and fragile and for these reasons were declined from surgery due to high surgical risk on renewed evaluation

- Crucial clinical characteristics related to cardiovascular sphere are missing: for example, coexisting cardiomyopathy, ventricular hypertrophy, valvular disease, arrhythmic burden and the presence of a pacemaker/defibrillator. Finally, also echocardiographic measures showed in table 1 are too sketchy; in this description a lot of relevant data are missing, as well as right ventricular dysfunction, diastolic function, estimated systolic pulmonary pressures.

- Evaluation of symptoms is rather weak; authors have to itemize how physicians have stated the lack of symptoms: they have to specified if they refer to anamnestically detected symptoms, since we can't find in the text any concern to a stress test. Due to the gradual progression of the stenosis and patient's adaptation, it could be difficult recognize symptoms onset; moreover, patients tend to deny the presence of symptoms or they could attribute them to the ageing process or other comorbidities. For these reasons in current guidelines exercise testing is recommended in physically active patients for unmasking symptoms and for risk stratification of asymptomatic patients with severe aortic stenosis; furthermore exercise stress echocardiography may provide prognostic information in asymptomatic severe aortic stenosis by assessing the increase in mean pressure gradient and change in LV function during exercise test.

- Baseline characteristics of study population should include the reason of admission to hospital

- Authors should explain how they perform follow-up evaluation; in this group of patients with severe aortic stenosis a close follow-up is mandatory; a scheduled visit could unmask symptoms onset and detect some clinical or bio-humoral changes and possible change therapeutic strategy. According to this porpoise one-year survival between two groups (age and gender matched) is similar: 89% in asymptomatic patients compared with 92% in patients referred to AVR.
Moreover, a subsequent echocardiogram could reveal an increase of the mean gradient, an incipient left ventricular dysfunction and make mandatory a surgical indication.

- Likewise authors recordered causes of death in the asymptomatic aortic stenosis group, they should list also causes of death in patient submitted to AVR.

- In competing risk regression analysis authors should add also troponin between variables at issue.

Minor points

- Title does not fully reflect main topics covered in the study; in particular patient characteristics are not been adequately investigated.

- Some sections have not a proper layout.

- Conclusions need to be reformulated.
